# Successful Case of Resection of the Knee Joint

**Published:** 1859-07

**Authors:** J. W. Freer

**Affiliations:** Prof. of Anatomy in Rush Medical College, one of the Surgeons to Mercy Hospital, etc.


ARTICLE III.

### SUCCESSFUL CASE OF RESECTION OF THE KNEE JOINT.

BY J. W. FREER, PROF. OF ANATOMY IN RUSH MEDICAL COLLEGE, ONE OF THE SURGEONS TO MERCY HOSPITAL, ETC.

CASE.—Mrs. Nichols, aged 30 years, of healthy appearance and good constitution, came under my notice in the spring of 1858, for disease of the right knee joint, which, according to the patient, was of two years' duration.

*History.*—The patient was not aware of ever having received an injury to the member. She related that the first indication of disease was slight pain and swelling in front and lower part of the joint. This rapidly increased, and in the course of a year a fistulous opening was established, communicating with the articular cavity. She had always managed to go about by the aid of crutches.

*Present appearance.*—Joint enormously swollen, partly from pressure of fluid within the articular cavity, as well as from the œdema without. Spontaneous dislocation of the leg outward, with semiflexion and anchylosis in front and below. A depressed cicatrix, making the situation of the former fistulous opening. The skin of natural color and temperature.

*Local symptoms.*—Severe pain on attempting to move the joint; no tenderness with moderate pressure; subject to paroxysms of severe pain while in the quiet condition, especially at night. The general health good.

In the month of August, 1858, the patient entered Mercy Hospital for treatment, although she did not come under my care until February, 1859, having in the mean time been under treatment by Dr. N. S. Davis, the result of which was a great diminution of the fluid in the articular cavity, although the limb remained the same in regard to immobility and deformity of the joint, as well as painfulness which had not appreciably diminished.

Believing it to be a suitable case for resection, and having carefully described the operation to the patient, with its possible and probable results, her assent was readily given; and on the 10th of February, in the presence of Drs. Powell, Hunt, Andrews and others, the operation was performed in the following manner:

*Operation.*—The patient being placed fully under the influence of chloroform, and placed in proper position, an incision was made, in outline like a horse shoe. The lesser portion corresponding with the tibia, an inch below the lesser tuberosity, the open sides with the pastero-lateral portion of the joint. The flap being dissected up, the ligamentum patello and lateral ligaments severed, the extremities of the bones were readily exposed by forcible flexion. Having removed the patella and carefully dissected away the soft parts from the bones to the required extent, excision was readily completed with the amputating-saw, section being made in the direction from before backward, the soft parts protected from the saw by inserting a strip of pasteboard. Two and a half inches of femur and one inch of the tabia were removed. Very slight hemorrhage ensued; two or three articular branches required the ligature. The wound was closed by interrupted suture, and the limb placed in a straight carved splint and foot board, with a long side splint attached firmly to the pelvis and foot.

*Morbid Anatomy.*—Articular cavity occupied with a small quantity of yellow cheese-like substance; synovial membrane much thickened and gelatinous in appearance; articular surfaces entirely denuded of cartilage, and presenting here and there ivory-like patches; portions were deeply excavated, as the inner condyle and glenoid surfaces of the tibia; cancellated stricture occupied with a yellow consistent substance, like strumous deposit; crucial ligaments, degenerated and scarcely recognizable; inner surface of patella denuded and carious.

The subsequent treatment was of the simplest character. No medicine was required after the first dose of morphine, at the time of the operation; no pain ensued of sufficient severity to indicate anodynes. During the three months of the patient's confinement, her appetite was uniformly good, and bodily functions regular. The wound, with the exception of two or three fistulous openings, healed by first intention. At the expiration of three months, the limb was dressed with pasteboard splints and paste bandages, and the patient permitted to go about on crutches.

*Present condition.*—At this time, 10th of June, about four months since the operation, we find the following condition: The discharge has entirely ceased; union has taken place, which is of sufficient firmness to maintain the immobility of the part, when the paste bandage is removed and the limb supported by the foot, or the patient directed to elevate the member without support. The shortening does not exceed two and a half inches. The limb is still sustained with the original pasteboard splints and paste bandage; a longitudinal division having been made in order to allow of its removal at any time, for the purpose of cleanliness or other convenience. Such being the present condition, we feel warranted in predicting a highly useful member in the future.

When the case has fully matured, we propose to give the final result.

